# Supplementary material for: Postoperative C-Reactive Protein Trend Is a More Accurate Predictor of Anastomotic Leak than Absolute Values Alone
Source: J Clin Med. 2025 Apr 24;14(9):2931. doi: 10.3390/jcm14092931 (PMC12072654; doi:10.3390/jcm14092931)
Supplement: Supplementary file 1 [file jcm-14-02931-s001.zip › jcm-3519234-supplementary.pdf]

Table S1. Concomitant operations performed with colectomy during index operation.

Surgery, n (%)	Control N=220	Anastomotic Leak N=9
Abdominal Wall Debridement	3.0 (1.4)	-
Hernia Repair	17.0 (7.7)	3.0 (33.3)
Diaphragm Resection	1.0 (0.5)	-
Fistula Takedown	13.0 (5.9)	-
Gastrectomy	3.0 (1.4)	-
Small Bowel Resection	10.0 (4.5)	1.0 (11.1)
Appendectomy	4.0 (1.8)	-
Liver Resection	9.0 (4.1)	1.0 (11.1)
Hepatic Artery Infusion Pump Insertion	3.0 (1.4)	-
Cholecystectomy	5.0 (2.3)	-
Splenorrhaphy	1.0 (0.5)	-
Adrenalectomy	1.0 (0.5)	-
Peritoneal Nodule Resection	2.0 (1.0)	-
Nephrectomy	2.0 (1.0)	-
Cystectomy or Bladder Repair	9.0 (4.1)	-
Prostatectomy	2.0 (1.0)	-
Salpingectomy	8.0 (3.6)	-
Hysterectomy	4.0 (1.8)	-
Hyperthermic Intraperitoneal Chemotherapy (HIPEC)	1.0 (0.5)	-
